# Human blood plasma catalyses the degradation of *Lycopodium* plant sporoderm microcapsules

**DOI:** 10.1038/s41598-019-39858-z

**Published:** 2019-02-27

**Authors:** Teng-Fei Fan, Michael G. Potroz, Ee-Lin Tan, Jae H. Park, Eijiro Miyako, Nam-Joon Cho

**Affiliations:** 1 School of Materials Science and Engineering, Nanyang Technological University, 50 Nanyang Avenue, 639798 Singapore, Singapore; 2 Department of Materials and Chemistry, Nanomaterials Research Institute (NMRI), National Institute of Advanced Industrial Science and Technology (AIST), Central 5, 1-1-1 Higashi, Tsukuba, Ibaraki 305-8565 Japan

## Abstract

Plant sporoderm are among the most robust biomaterials in nature. We investigate the erosion of *Lycopodium* sporoderm microcapsules (SDMCs) triggered by human blood plasma. Dynamic image particle analysis (DIPA), field emission scanning electron microscopy (FESEM) and Fourier transform infrared (FTIR) spectroscopy demonstrate the degradation events, suggesting bulk erosion as the dominant mechanism for SDMCs fragmentation in human blood. These results should prove valuable in discerning the behaviour of SDMCs in potential biological applications.

## Introduction

There has been considerable interest in developing robust biocompatible materials for a range of biomedical and industrial applications^1–6^. Plant sporoderm microcapsules (SDMCs) have emerged as a promising biomaterial for such applications^7–11^. SDMCs can be obtained by the extraction of plant sporoderm, the outer layer of plant spores or pollen^8,12,13^. Most spore/pollen cell cytoplasm, which is rich in nutrients and active substances including proteins, vitamins, carbohydrates and pigments, can be extracted^8,12,14^. These SDMCs are naturally produced by plants and are abundant in large quantities^15–17^. In addition, they are mainly composed of sporopollenin, an extremely robust mix of biopolymers that are resilient to harsh chemical (e.g., a variety of strong acids and alkalis) and physical conditions (e.g., high pressure or mechanical stress)^12^. Due to these properties, the sporopollenin was described as the “toughest natural biopolymer” and considered “practically indestructible”^11,18^. There is wide variation in the physiochemical properties of plant sporoderm^19–21^, which feature highly monodisperse microcapsules characterised by exquisite and unique three-dimensional (3D) architectures^21–23^. From the precious reports, sporopollenin was considered harmless to human health and was applied on skin without irritation, and was reported safe for consumption (LD_50_ *rat* > 2000 mg/Kg) and injection in the blood stream^8^. Clinical trials were also performed to verify that sporopollenin particles in the blood system were degradable^8,9^. The SDMCs can be used as microcapsules or as micron-sized particles with functionalizable surfaces. The SDMCs can be filled with polar and non-polar materials via multi-directional nano-diameter sized pores. Furthermore, highly biocompatible features of naturally produced SDMCs are valuable for designing functional materials. In fact, given the unique characteristics of SDMCs, their versatility has been explored in micro-encapsulation^8,16^, heavy metal binding^24^, catalysis^25,26^, biosensors^27^ and as scaffolds for producing porous 3D structures^11,28^. A wide variety of chemical compounds, biotherapeutics, foods and cells have already been encapsulated in spore-based capsules^29–33^.

In past decades, plant scientists have studied plant sporoderm chemistry extensively. It was widely accepted based on chemical and molecular genetic analysis that sporopollenin contains phenolics and polyhydroxylated aliphatics that are covalently coupled by ether and ester bonds^34^. However, the chemical structure of sporopollenin requires further study. In recent years, Fourier transform infrared (FTIR) spectroscopy has proven to be an ideal approach to spore/pollen chemistry analysis without any sample pre-treatment, and it has also proven to be useful to distinguish specific species^35,36^. Indeed, the IR spectrum of pollen can be roughly divided into specific regions containing signatures of lipids, proteins, carbohydrates and grain wall biopolymers called sporopollenins. As features related to these compounds are responsible for the lion’s share of phenotypic attributes, FTIR spectroscopy is, therefore, an excellent tool for species specific and biochemical analysis of pollen.

Although extremely robust, several methods for sporopollenin degradation have been reported, including ozonolysis^37^, acetolysis^38^, photocatalytic reactions^39^ and redox reactions with hydrogen peroxide^40^, peroxidase and catalase^40^. However, much of the researches pertain to harsh conditions that bear no correlation to biological systems – a particularly important point for potential biological applications of sporopollenin-based microcapsules such as drug delivery considering its biocompatibility, renewability and low cost. In addition, the natural Lycopodium and the extracted sporopollenin exine capsules (SECs) can withstand the harsh environment of the stomach, which would facilitate the oral administration and controlled drug release into the gastrointestinal (GI) tract^8,9,13,16,17,41–43^. It was recently reported that SECs composed mainly of sporopollenin, could permeate through intestinal walls and decompose in the blood to release their contents within 30–60 mins^9,17,25,44^. Further, it was suggested that enzymes present in human plasma would play a role in the degradation of SECs^9,45^. However, the exact mechanism through which SECs enter the blood stream and subsequently decompose is still unclear.

In this work, we investigate the degradation of SDMCs in human blood plasma. SDMCs from *Lycopodium clavatum* were extracted by acid hydrolysis using a previously reported methodology^12^. Biodegradation experiments were conducted by incubating extracted SDMCs in human blood plasma at 37 °C. The degradation of SDMCs was then characterised at time points of 10 min, 30 min, 1 h, 12 h and 96 h, sequentially. Morphological characterisation was performed with dynamic image particle analysis (DIPA)^12,46^ and field emission scanning electron microscopy (FESEM). Further, we analysed the equivalent spherical diameter (ESD), aspect ratio and edge gradient of SDMCs for investigating structural changes of SDMCs in human blood plasma. The analysis was performed at time points up to 96 h. SDMC′s chemistry was also characterised via FTIR spectroscopy.

## Materials and Methods

### Materials

Natural *L*. *clavatum* spores and other chemicals were purchased from Sigma-Aldrich (St. Louis, MO, USA). Fresh normal blood plasma was purchased from Axil Scientific Pte Ltd. (Singapore), and all of the cells were separated by centrifuge at 2000 g for 15 min. Simulated body fluid (SBF) was prepared according to the literature^45^.

### Extraction of SDMCs from *Lycopodium clavatum*

The SDMCs were extracted using the acid hydrolysis method, as previously reported^16^.

### Degradation treatment of SDMCs

The SDMCs (40 mg) were suspended in human plasma (0.6 mL) and incubated at 37 °C with a rotating speed of 220 rpm for 10 min, 30 min, 1 h, 12 h and 96 h. The SDMCs were then collected by centrifugation at 7000 rpm for 15 min, washed with SBF (×5) and deionised water (×5) and then left to dry in a freeze-dryer.

### Dynamic image particle analysis (DIPA)

DIPA was performed in a FlowCAM (FlowCamVS, Fluid Imaging Technologies, Maine, USA) equipped with a 200-μm flow cell (FC-200) and a 20× magnification lens (Olympus®, Japan). Untreated SDMCs and human plasma-treated sample solutions with a concentration of 2 mg mL^−1^ were primed manually into the flow cell (a pre-run volume of 0.5 mL) and analysed with a flow rate of 0.1 mL min^−1^ and a camera rate of 14 frames sec^−1^. The particle count for each measurement was fixed at 10,000 particles, and three separate measurements were performed. From each measurement, 500 well-focused spores were segregated based on edge gradient ordering and manual processing. The instrument was calibrated using standard polystyrene microspheres (50 ± 1 μm), and the collected data were depicted as histograms with a spline line curve.

### Fourier transform infrared (FTIR) spectroscopy

FTIR measurements were performed on a PerkinElmer (Seer Green, Buckinghamshire, UK) Spectrum with a diamond cell ATR accessory. The reflectance infrared spectra were collected using the PerkinElmer Spectrum software at 16 scans per sample. Background spectra were collected prior to each set of six replicates and automatically subtracted from each replicate using a sample-free set up. The spectra were generated in the 4000–600 cm^−1^ spectra range, with a spectral resolution of 4 cm^−1^. Baseline corrections were carried out using the Spectrum 10 software. Following baseline correction, each spectrum was standardised as previously reported using the equation $({\rm{x}}-\bar{{\rm{x}}})/\sigma$, where x is the absorbance value, $\bar{{\rm{x}}}$ is the spectrum arithmetic mean, and σ is the spectrum standard deviation. Peak height was measured by taking the maximum value within a given range (Table 1). Peak ratios between different peaks were also calculated to remove the potential impact of differing sample thicknesses on the absolute absorbance values.

**Table 1:** Peak assignments for absorbance peaks in the FTIR spectra. The assignment and interpretation were based on the previous literature^34,35,38,46,51^.

Wavenumber (cm^−1^)	Assignment	Interpretation
3384	νO-H	Hydroxyl
2925	ν_as_C-H	Methyl group (lipid, sporopollenin)
1745	νC=O^a^	Ester group (lipid, sporopollenin)
1707	νC=O^b^	Carboxyl (sporopollenin)
1516	νC=C	Aromatic (sporopollenin)
1442	δCH_3_	Methyl groups (Lipid, sporopollenin)
1140	νC-O^a^	Carbohydrate
993	νC-O^b^	Carbohydrate
815	δC-H	Aromatic (sporopollenin)

### Scanning electron microscopy (FESEM)

SEM images were taken with an FESEM 7600 F (JEOL, Japan). Samples were sputter-coated with gold at a thickness of 20 nm using a JFC-1600 (JEOL, Japan) (20 mA, 80 s), and images were recorded with an acceleration voltage of 5.00 kV at different magnifications to provide morphological information.

## Results and Discussion

### DIPA

**Figure 1:**
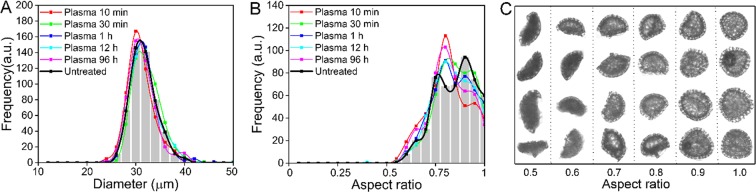
Morphological properties of *Lycopodium* SDMCs before and after degradation treatment in human plasma as characterised by DIPA. (**A**) Diameter distribution of SDMCs; (**B**) Aspect ratio of SDMCs; (**C**). Representative *Lycopodium* SDMCs with different aspect ratios. The plots are representative graphs obtained by the spline curve fitting of histogram data from 500 well-focused particle images after triplicate measurements (n = 3).

The results of the DIPA measurements are presented in Fig. 1. In terms of the size distribution (Fig. 1A), the majority of particles were in the range of 22–40 μm, with no significant differences between the untreated samples and the incubated samples. The mean ESD was 30.65 ± 2.71 µm for the untreated samples and 30.83 ± 2.97 µm for the 96 h degradation samples (Fig. 1A).

In terms of the aspect ratio (Fig. 1B), untreated SDMCs demonstrated two major populations at ca. 0.75 and 0.9. Upon subsequent incubation in plasma, the portion of SDMCs with an aspect ratio of ca. 0.9 decreased, with the main population of particles at an aspect ratio of ca. 0.8. This change in morphology suggests possible degradation with plasma incubation.

The morphological changes of the SDMC samples were closely examined categorically (Fig. 1C). The untreated *Lycopodium* SDMCs were seen to be uniform-sized as trihedral pyramids, with aspect ratios between 0.7 and 1.0. However, broken SDMCs were seen to be altered into irregular pieces with aspect ratios between 0.5 and 0.6. Hence, it can be inferred that the observed population shift in terms of aspect ratio following blood plasma incubation is a result of partial degradation rather than complete breakage, which would agree with previously reported scanning electron microscopy images of partially degraded SECs that were treated in rat blood for 30 min^17^.

**Figure 2:**
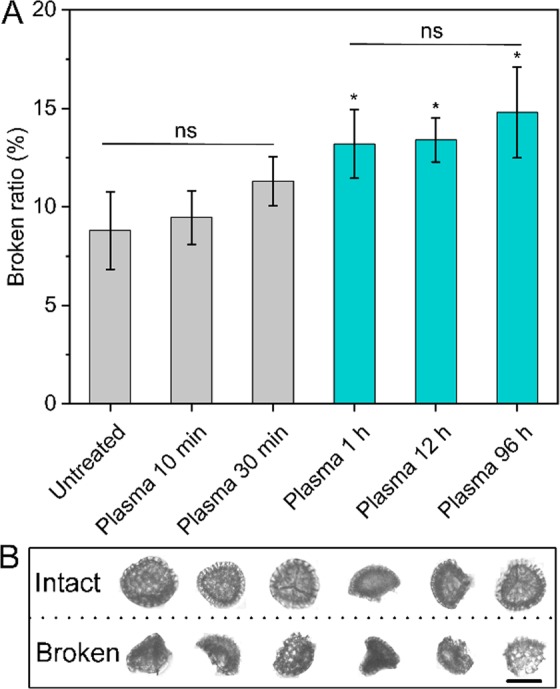
Integrity analysis of *Lycopodium* SDMCs before and after human blood plasma treatment as measured by DIPA. (**A**) Broken ratio of *Lycopodium* SDMCs before and after blood plasma treatment; (**B**) Representative DIPA images of intact and broken *Lycopodium* SDMCs. Data shown are the averages of triplicate measurements with standard deviation (n = 3). The scale bar is 20 μm.

Moreover, DIPA analyses demonstrate definitive morphological changes following incubation in human blood plasma (Fig. 2). When inspecting the integrity of individual particles, it was found that the number of broken SDMCs progressively increased upon exceeding 30 mins of blood plasmsa incubation (Fig. 2A). After 96 h incubation, 14.8% of SDMCs were damaged, which is a 1.5-fold increase from that observed for untreated samples (8.8%). Considering the sporopollenin’s high resistance to mechanical stress^11,18^, the observations suggest that the increased fragility of SDMCs are resulted by partial degradation in human blood plasma. Interestingly, despite evidences of degradation and breakage of the particles resulting in large fragments, a corresponding increase in smaller fragments were not apparent which is evident in the lack of change in size distribution and the minimal shift in aspect ratio as measured by DIPA (Fig. 2B).

### FTIR

**Figure 3:**
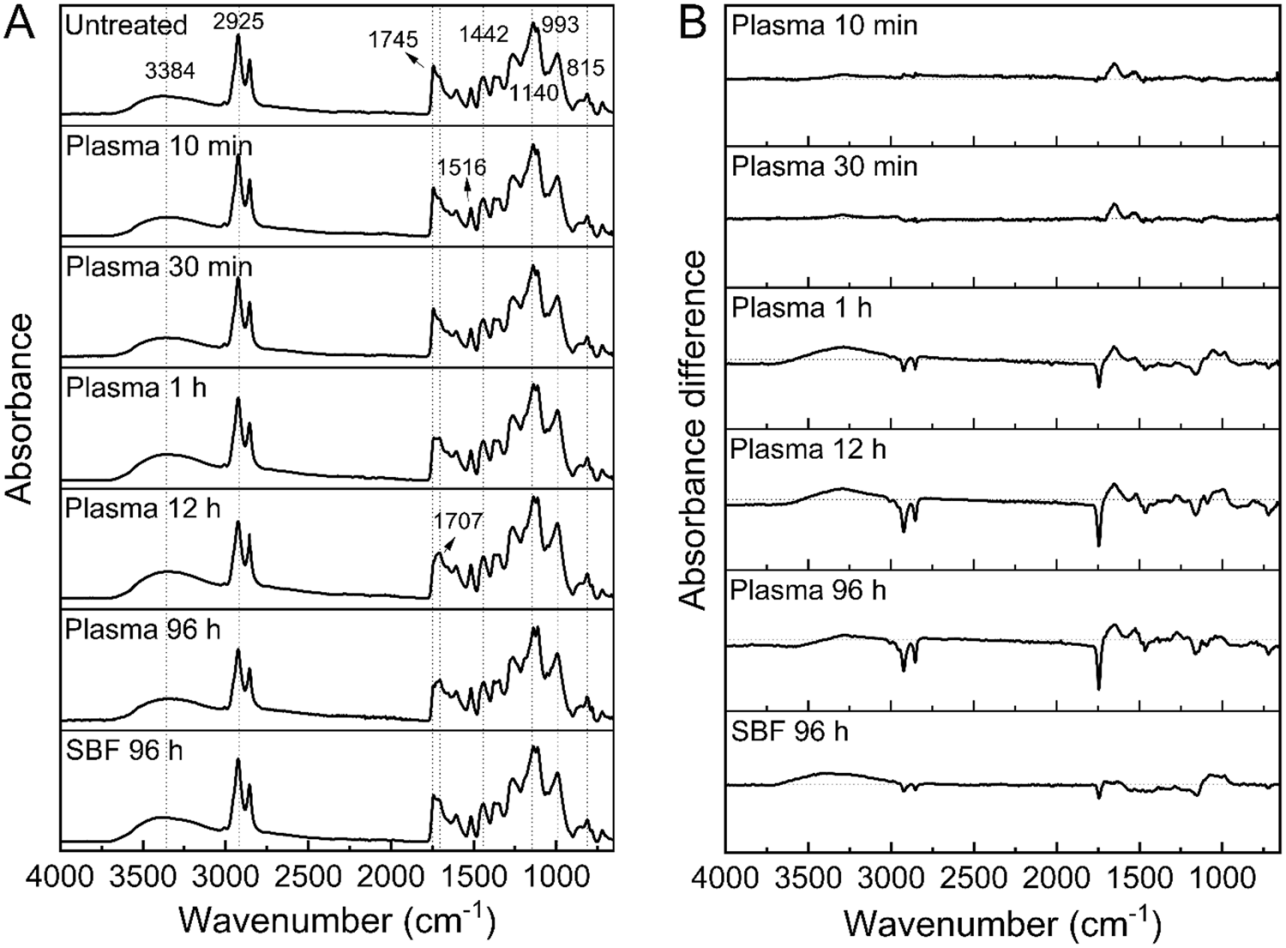
FTIR spectrum and the absorbance difference spectrum for *Lycopodium* SDMCs before and after degradation treatment in plasma (10 min, 30 min, 1 h, 12 h, and 96 h) and simulated plasma fluid (96 h).

To better understand the underlying mechanism of degradation, FTIR spectroscopy was used to characterise changes in the chemistry of the SDMCs. As presented in Fig. 3 and Table 1, the following FTIR peaks from the untreated *Lycopodium* were assigned: 3384 cm^−1^ (stretching vibration of O-H bond); 2925 and 2850 cm^−1^ (aliphatic vibrations); 1745 and 1707 cm^−1^ (ester and carboxyl groups, stretching vibration of C=O bond); and 1140 and 993 cm^−1^ (sporopollenin and/or carbohydrate bonds, *e*.*g*. cellulose and amylose). These values correlate well with those found in the literature^35,39,47^. Samples treated with human blood plasma for 10 min and 30 min showed only slight differences in their spectra compared to untreated samples, as shown by small positive shifts at 1651cm^−1^ and 1531 cm^−1^. However, these wavenumbers are not significant for the SDMCs, with no clear trend observed over the various time points in this region of the spectra.

In marked contrast, significant changes were observed in the FTIR spectra after 1 h of blood plasma incubation. The sharp absorbance peak observed at 1745 cm^−1^ flattened, and absorbance peaks at 2925, 2850, 1745 and 1442 cm^−1^ decreased in height. Interestingly, all of these peaks are associated with lipids, which suggests that the degradation of the SDMCs in human blood plasma occurs with the degradation of its lipid components. Similar profound changes were observed after 12 h incubation. However, no significant changes were observed between 12 h and 96 h. Overall, these changes indicate the degradation of the SDMCs, with most of the degradation occurring within 12 h. Observed fluctuation at 3384 cm^−1^ was attributed to the destruction and reformation of hydroxyl groups.

To discern the specific effect of enzymes on SDMC degradation, simulated blood fluid (SBF) without enzymes was used for incubation instead of the human blood plasma treatment^48,49^. It was found that after 96 h incubation in SBF the magnitudes of changes were extremely diminished, which confirms the functional role of human blood plasma in SDMCs degradation. This correlates well with the literature that reports the physical degradation of SECs in human/rat blood and plasma but not in SBF or serum^9,17^.

**Figure 4:**
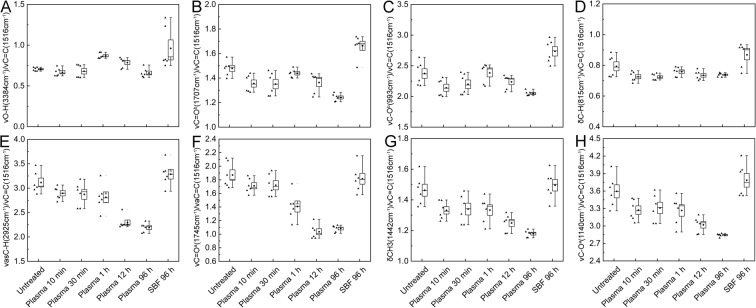
Box plot of the FTIR peak height ratios among hydroxyl, carboxyl, carbohydrate, aromatic, and aliphatic peaks in *Lycopodium* SDMCs before and after blood plasma incubation. The νC=C peak (1516 cm^−1^) was set as the internal standard peak. In each box plot, the dot indicates the median, and the whiskers at both ends are the highest and lowest points within 1.5 standard deviations.

Although it is difficult to precisely quantify the chemical changes in SDMCs due to the complex nature of sporopollenin, we were able to decypher the peak response of the specific functional groups (hydroxyl, carboxyl, carbohydrate, aromatic, and aliphatic groups) before and after blood plasma treatment (Fig. 4). Using this method, the potential impact of different sample thicknesses on the absolute absorbance values can be eliminated, giving a better quantitative approach to evaluating the chemical changes^38^. Based on the absorbance difference spectrum (Fig. 3B), the 1516 cm^−1^ peak (aromatic) was shown to be relatively stable across incubation times (showing the least variance in peak height). This peak was therefore chosen as the internal standard and peaks at 3384 cm^−1^ (hydroxyl groups); 2925, 1745 and 1442 cm^−1^ (ester group); 1140 and 993 cm^−1^ (carbohydrates); and 1707 and 815 cm^−1^ (sporopollenin component) were chosen as the reference peaks for the corresponding functional groups to analyze the chemical changes.

After a 96 h incubation of SDMC sample in blood plasma, the ν_as_C-H(2925 cm^−1^)/νC=C(1516 cm^−1^), νC=O^a^(1745 cm^−1^)/νC=C(1516 cm^−1^), δCH_3_(1442 cm^−1^)/νC=C(1516 cm^−1^) and νC-O^a^(1140 cm^−1^)/νC=C(1516 cm^−1^) ratios were seen to decrease significantly (1.4-, 1.9-, 1.2- and 1.3-fold less than that of the control, respectively).

In contrast, νC=O^b^(1707 cm^−1^)/νC=C(1516 cm^−1^), νC-O^b^(993 cm^−1^)/νC=C(1516 cm^−1^) and δC-H(815 cm^−1^)/νC=C(1516 cm^−1^) were relatively stable. These results highlight previous indications that the lipid component of SDMCs is the most unstable when subjected to incubation in human blood plasma^8,17,37^. Meanwhile, the aromatic components of SDMCs are seen to be the most stable. When incubated in enzyme-free SBF, the peak ratios showed no significant differences from those observed for the control, again indicating the importance of enzymes in the degradation process. Bearing in mind that SDMCs were extensively used as drug carrier, there needs to be more awareness of the byproducts, especially when it is being delivered into the human or animal body. In addition, the chemical changes of SDMCs circulating in the blood system should be thoroughly studied in the future.

### FESEM

**Figure 5:**
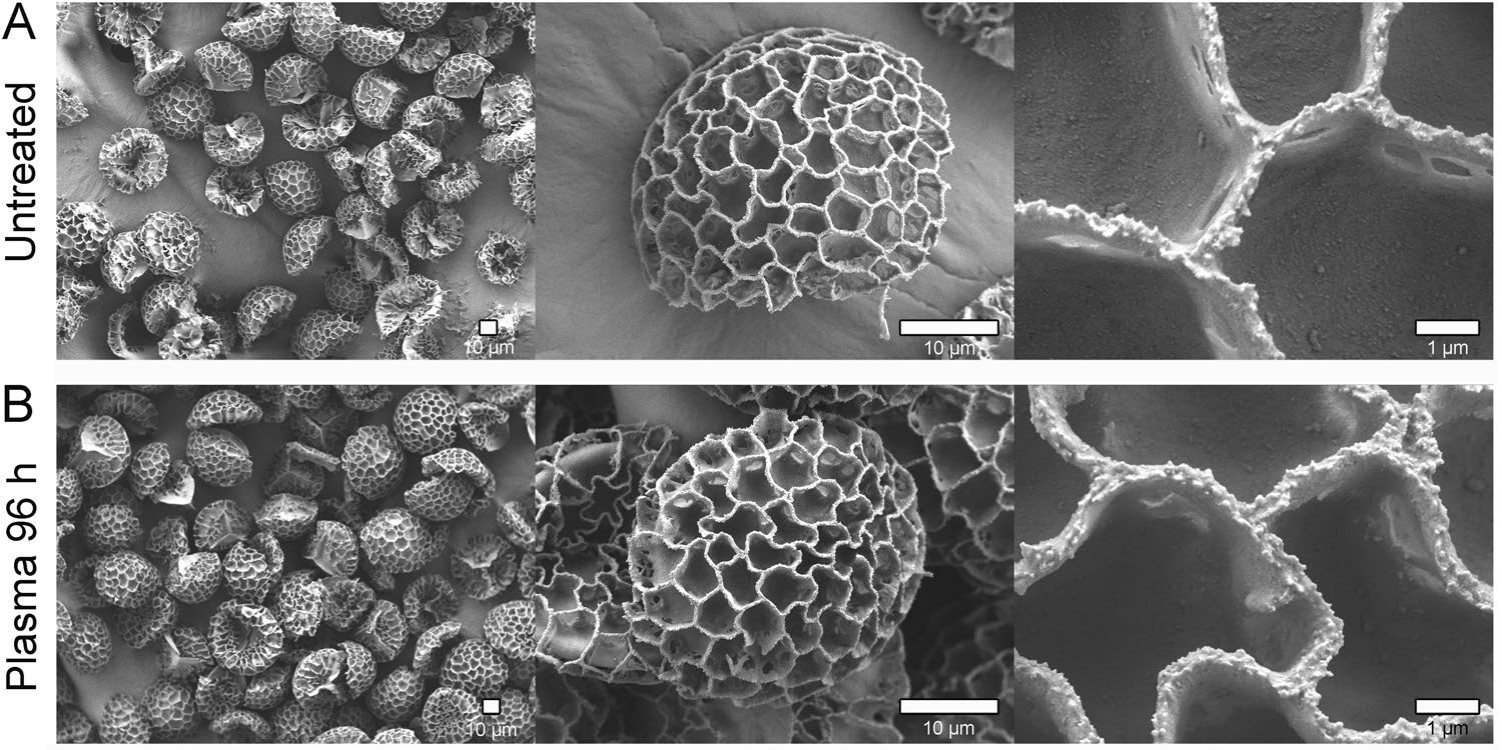
Surface morphology characterisation of *Lycopodium* SDMCs before and after blood plasma treatment performed by FESEM. (**A**) Representative images of untreated *Lycopodium* SDMCs at different magnifications (500×; 3000×; 15000×); (**B**) Representative SDMCs after 96 h blood plasma treatment.

Finally, the surface morphology of SDMCs before and after blood plasma treatment was characterised by FESEM (Fig. 5). No substantial surface degradation of the microstructures was observed after incubation for 96 h in human blood plasma. Contrast to the claims that the degradation of sporopollenin proceeds through surface erosion and bulk erosion^50,51^, the mechanism of *Lycopodium* SDMC′s degradation in human blood appeared to proceed through bulk erosion rather than surface erosion. This is deduced by the minimal changes observed in surface morphology from DIPA. Furthermore, considering that the SDMCs consist of a homogenous matrix of biological components, it is possible to predict degradation of ester groups within this network as seen by the FTIR, which would lead to bulk erosion.

## Conclusions

In summary, *Lycopodium* SDMCs were shown to partially degrade in human blood plasma as demonstrated by both physical and chemical data. While DIPA showed an increase in broken particles, FTIR demonstrated the erosion of lipid components of SDMCs. Herein, we highlight the chemical changes by the FTIR data, which was missing in the previous study which merits more investigations especially when it is considered for drug-delivery applications. The biosafety of byproducts from SDMC degradation should also be studied in the future. Based on DIPA and FESEM analyses, bulk erosion appeared to be the main mechanism behind SDMC degradation. Overall, with the growing promise of SDMCs as functional biomaterials for a variety of applications, we believe that these findings will prove valuable in future evaluations of its biosafety and efforts to discern their behaviour in biological conditions.
